# Intellectual Abilities of Children with Narcolepsy

**DOI:** 10.3390/jcm9124075

**Published:** 2020-12-17

**Authors:** Marine Thieux, Min Zhang, Agathe Marcastel, Vania Herbillon, Anne Guignard-Perret, Laurent Seugnet, Jian-Sheng Lin, Aurore Guyon, Sabine Plancoulaine, Patricia Franco

**Affiliations:** 1 Pediatric Sleep Unit, Department of Pediatric Clinical Epileptology, Sleep Disorders and Functional Neurology, Hôpital Femme Mère Enfant, Hospices Civils de Lyon, 69500 Lyon, France; marine.thieux@chu-lyon.fr (M.T.); agathe.marcastel@chu-lyon.fr (A.M.); vania.herbillon@chu-lyon.fr (V.H.); anne.guignard-perret@chu-lyon.fr (A.G.-P.); aurore.guyon@chu-lyon.fr (A.G.); 2 INSERM, U1028, CNRS, UMR5292, Lyon Neuroscience Research Center, 69500 Lyon, France; min.zhang@etu.univ-lyon1.fr (M.Z.); laurent.seugnet@inserm.fr (L.S.); jian-sheng.lin@univ-lyon1.fr (J.-S.L.); 3 Université de Paris, CRESS, INSERM, INRAE, 75004 Paris, France; sabine.plancoulaine@inserm.fr

**Keywords:** cognition, children, REM, intelligence quotient, narcolepsy, obesity, obstructive sleep apnea

## Abstract

High cognitive functioning could be a protective factor for school difficulties, behavioral and mood impairments in children with narcolepsy. To investigate this factor, we studied the intellectual abilities of 74 children with narcolepsy (43 boys, 11.7 years old at diagnosis, 91% of cataplexies, 64% obese, 100% HLA positive for DR-DQB1*06:02). All children underwent a one-night polysomnography followed by Multiple Sleep Latency Tests, an evaluation of intelligence quotient (IQ), and filled standardized questionnaires. Thirty-eight percent had high potentialities (HP defined by IQ > 130) and 48% had school difficulties. Using non-parametric tests, we found that HP children reported less difficulties at school and tended to have less impulsivity, conduct, and learning disorders than those without HP. They also tended to be less obese and had less desaturation. Using a multivariate regression analysis, we found an association between the REM sleep percentage and the IQ. REM sleep could be involved in the dynamic changes contributing to the equilibrium of intellectual functioning. This study highlights that despite their frequent school difficulties, narcolepsy per se is unlikely to be a cause of intellectual disability in children. Prompt diagnosis and management of comorbidities such as obesity and obstructive sleep apnea (OSA) could improve cognitive and school performances in these children.

## 1. Introduction

Narcolepsy is a rare disabling sleep disorder characterized by excessive daytime sleepiness (EDS) and abnormal rapid eye movement sleep (REM) manifestations including cataplexy (sudden loss of muscle tone triggered by emotions), hypnagogic hallucinations, sleep paralysis, and disturbed nocturnal sleep [1,2]. Narcolepsy with cataplexy is caused by a deficiency of hypocretin-1 (also called as orexin) peptides released from the dorso-lateral hypothalamic neurons (also called narcolepsy type 1 or hypocretin deficient) [3,4]. Hypocretin is involved in the regulation of sleep–wake cycle [4], in cognitive functioning [5], and in the processes promoted by the limbic system (i.e., involved in stress, reward, emotions, and motivation control) [6]. If the etiology of hypocretin cells’ death is still unknown, multiple factors have been implicated: genetic, autoimmune, and environmental [2,3,4].

More than half of the patients have a disease onset prior to 18 years old [7]. At diagnostic time, the clinical picture is quite different in children compared to adults [8]. Children present more frequently obesity, night eating, parasomnia, sleep talking, sleep drunkenness and Attention Deficit Hyperactivity Disorders’ symptoms than adults [8]. Although depressive feelings affect similarly children and adults, they have a higher impact on adults’ quality of life [8].

Compared to healthy children, those with narcolepsy have more school difficulties, grade repetition, and absenteeism [9,10]. School difficulties might be related to cognitive impairment, which has also been described in patients with narcolepsy. Historically, these cognitive alterations were mainly based on subjective complaints or clinical observations [7]. Then, several studies in adults with narcolepsy assessed the existence of objective cognitive disorders. Some of these studies suggested that while patients may attain high performances in several neuropsychological subdomains, they can have difficulties in areas such as working memory, executive functioning, reward processing, and decision making [11,12,13]. In adults with narcolepsy with cataplexy, attention and executive disorders, especially for long and complex cognitive tasks, have been reported [12,14,15,16,17,18] with a deleterious impact on quality of life.

Very few studies have addressed this issue, usually in small groups of children with narcolepsy [11,19,20]. Although the authors found normal intelligence quotient (IQ) abilities, some of them reported that those with low intellectual abilities had more behavioral and psychiatric pathologies [11]. Indeed, previous studies have suggested that mid to high intellectual functioning acts as a protective factor in terms of recovery, adaptation, and psychological states in people with psychiatric and neurological conditions and in children with chronic disorders [14,21]. In adults with narcolepsy, a mid-to-high IQ is associated with less cognitive impairment and less mood disorders [14]. Such an assessment remains to be done in children with narcolepsy.

Therefore, the aims of the present study were to describe the intellectual abilities in a large group of children with narcolepsy and to characterize which factors could influence these abilities. Another objective was to investigate whether these abilities constitute a protective factor for school difficulties, behavioral, and mood impairments in these children.

## 2. Material and Methods

### 2.1. Patients

All children presenting idiopathic narcolepsy with or without cataplexy (age < 18 years old) seen in our national reference center for narcolepsy between 2010 and 2019 were included in this retrospective study (*n* = 74). This study was approved by the French National Information science and Liberties Commission (HCL CNIL register no 19-087).

### 2.2. Procedure

#### 2.2.1. General Data Collection

All children (and parents) had a systematic interview with a pediatric sleep specialist (PF, AGP) in order to assess the sleep disorders and to control the wake–sleep schedule before the recording night. In addition, parents were asked about the presence of school difficulties for her/his child, with a status of yes/no to categorize the answer. Socioeconomical level (SEL) was evaluated based on the parents’ occupational level. Data about narcolepsy history, current cataplexy symptoms, hypnagogic hallucinations, and sleep paralysis (with a yes/no answer for all these characteristics) were also collected by the pediatric sleep specialists.

#### 2.2.2. Diagnostic Procedure

The sleep and wake monitoring procedure included: (1) a complete clinical examination; (2) a polysomnography (PSG) from 8 pm to 7 am; (3) followed by 4 or 5 standard Multiple Sleep Latency Tests (MSLT) at 9 am, 11 am, 1 pm, 3 pm, and 5 pm, which were terminated after 20 min if no sleep occurred, and after 15 min asleep if sleep occurred [22]. The PSG included 8 EEG referenced to the mastoids according to the 10–20 system, 2 electro-oculograms, 1 levator menti surface electromyography, nasal pressure through cannulae, thoracic and abdominal belts, 1 ECG, transcutaneous oximetry during the night. Sleep stages, arousals, and respiratory events were scored visually according to standard pediatric criteria [22]. Total sleep time (TST), sleep and rapid eye movement (REM) latency, sleep efficiency, duration and percentage of stage 1 (N1), stage 2 (N2), stage 3 (N3), and REM sleep, arousal index, obstructive apnea hypopnea index (OAHI), index of desaturation ≥ 3%, and minimal saturation were collected.

#### 2.2.3. Criteria for Idiopathic Narcolepsy

All patients met the criteria for idiopathic narcolepsy [23], including: (1) complaints of excessive daytime sleepiness for at least 3 months; (2) symptoms not better explained by other medical or psychiatric disorders; (3) absence of secondary narcolepsy; (4) presence of clear-cut cataplexy; and/or (5) mean sleep latency on Multiple Sleep Latency Test (MSLT) lower than 8 min and/or two or more sleep-onset REM periods (SOREM) [22]. Indeed, the presence of two or more sleep-onset REM sleep periods is a more specific finding than the mean sleep latency of less than 8 min [24]. HLA-DR-DQB1*06:02 genotyping was performed in most patients (*n* = 71). Hypocretin-1 was also determined in 40 patients, in duplicate, from cerebral spinal fluid (CSF) samples as previously described [25]. Low CSF hypocretin-1 levels were considered when <110 pg/mL, intermediate when ≥110 and 200≤, and normal when >200.

#### 2.2.4. Anthropometric Measurements

Height and weight were obtained in each child, and the body mass index (BMI = weight/height2) was calculated. The BMI z-score representing a measure of weight, adjusted for height, sex, and age, relative to a smoothed reference distribution, was computed. Overweight was defined when the BMI z-score was ≥1.6 and ≤1.99 and obesity when the BMI z-score was ≥2 [26].

#### 2.2.5. Questionnaires

Four questionnaires were filled in by parents or by children under parental supervision: (a) The Adapted Epworth Sleepiness Scale for children in which the item “falling asleep while in a car stopped in traffic” was replaced by “falling asleep at school” assesses the risk of falling asleep in 8 daily-life situations estimated on a 4-point Likert scale. The total score is the sum of the scores for the 8 items: a higher score represents greater sleepiness and the pathological threshold is higher than 10 [27]; (b) The Insomnia Severity Index (ISI) [28] assess insomnia severity via 7 items scored on a 5-point Likert scale. The higher the total score, the more severe symptoms. The total score is considered pathological when it is higher than 10; (c) The Child Depression Inventory (CDI) assesses depressive symptomatology using 27 items based on a 3-point Likert scale, its abnormal cut-off score is 16 or higher [29]; and; (d) The Revised Conners Parents Rating Scale assesses behavioral and attention disorders using 48 items. This version is divided in 6 components (i.e., conduct, learning, psychosomatic, impulsivity, anxiety, and hyperactivity), and each component is standardized within age groups. Moderate to severe symptoms were defined with a cut-off above 65, severe symptoms were defined with a cut-off above 75 [30].

#### 2.2.6. Neuropsychological Evaluation

The children’s psychometric assessments were performed by experienced neuropsychologists (AM, VH, MT) using the Wechsler Intelligence Scale for Children (WISC IV) [31]. The total IQ is the combination of the following 4 indexes: verbal comprehension index (VCI), perceptual reasoning index (PRI), working memory index (WMI), and processing speed index (PSI). Each score is standardized within each age group: the mean is 100 and the standard deviation is 15. The heterogeneity of the cognitive profile is defined by an absolute difference between VCI and PRI ≥ 15, which is known as the Significant Verbal Performance Discrepancy (SVPD). When the cognitive profile is heterogeneous, the IQ is not calculated, and the General Aptitude Index (GAI), calculated from VCI and PRI, can be an alternative to IQ. GAI is less influenced by processing speed and working memory.

An IQ ≥ 130 is commonly accepted to define high potential (i.e., more than 2SD from the mean of the normal distribution) [32,33]. Intellectual disability is defined according to the WISC criteria by IQ ≤ 70. Based on their WISC scores, children were divided into two groups, those with high potential (HP) with an IQ, GAI, VCI, and/or PRI ≥ 130 and those without HP (No-HP). Before 2015, the WISC IV test was performed after the PSG diagnostic evaluation, and the children were already under a psychostimulant treatment during the neuropsychological test. After 2015, the WISC IV was performed during the diagnostic procedure without any treatment.

#### 2.2.7. Statistical Analysis

Statistical analyses were conducted by M.T. using the R software (version 3.6.3, Vienna, Austria) [34].

Continuous measures were expressed as median and range. Dichotomous and polytomous measures were expressed as *n* and percentage. Comparison between groups (HP vs. No-HP) was performed using Wilcoxon tests for continuous measures, Fisher’s exact test for dichotomous measures, and the Chi2 test for polytomous measures. A false discovery rate (FDR) correction for multiple comparisons was used. Statistical significance value was set to a *p*-value below 0.05.

In a recent study, our team found that children with HP had more REM sleep than children without HP [35]. In order evaluate this assertion in children with narcolepsy, a Pearson correlation between IQ and REM sleep was computed because of the normality of the distributions assessed by the Shapiro–Wilk test. Therefore, we computed a multivariate linear model assessing the association between IQ and percentage of REM sleep adjusting for BMI-z score, index of desaturations ≥ 3%, and group (HP vs. No-HP). These factors were chosen according to the results of the bivariate analysis in order to understand which factors may have an influence on IQ.

## 3. Results

The cohort was composed of 74 children (43 boys) with a median age of 11.7 years at diagnosis (Table 1 and Table 2). Thirty-two (43%) were under 11 years old at diagnosis. Using the BMI z-score, 47 were obese (64%) and 7 were overweight (10%; Table 1). Thirty-four children had school difficulties (48%) (Table 1). Median age at disease onset was 9.6 years old with a median diagnosis delay of 1.4 years. Sixty-seven children had cataplexies (91%), 13 had sleep paralysis (18%), and 29 had hypnagogic hallucinations (39%). All the patients were positive for HLA-DR-DQB1*06:02 (Table 2). All the children evaluated had low hypocretin-1 levels, except one who had an intermediate level. The girl with the intermediate level was 11 years old at diagnosis, with a diagnosis delay of 4.5 years, cataplexies and a median sleep latency of 10 min and 3 SOREM on MSLT.

Concerning PSG characteristics, median OAHI was 0.5/h with a minimal saturation value of 93%. On the MSLT, children had a median sleep latency of 2.5 min with 4 SOREM (Table 3). Pathological scores were found on the Epworth, ISI, and CDI scales in 96%, 71%, and 21%, respectively. The Conners median total score was 18 (Table 4). Forty-three children (58%) were under treatment at the time of the WISC evaluation: 30 children underwent a monotherapy (19 with Modafinil, 10 with Methylphenidate, and 1 with Mazindol), 13 children underwent a bi-therapy (8 with Methylphenidate and Venlafaxine, 4 with Methylphenidate and Modafinil, and 1 with Modafinil and Venlafaxine; Table 5). The WISC median scores were dispersed as follows: 118 for VCI, 109 for PRI, 103 for WMI, 100 for PSI, 115 for GAI, and 114 for IQ (Table 5). There were 28 children with HP (38%) and there were 13 children with a heterogenous IQ (18%) measured by SVPD.

**Figure 1:**
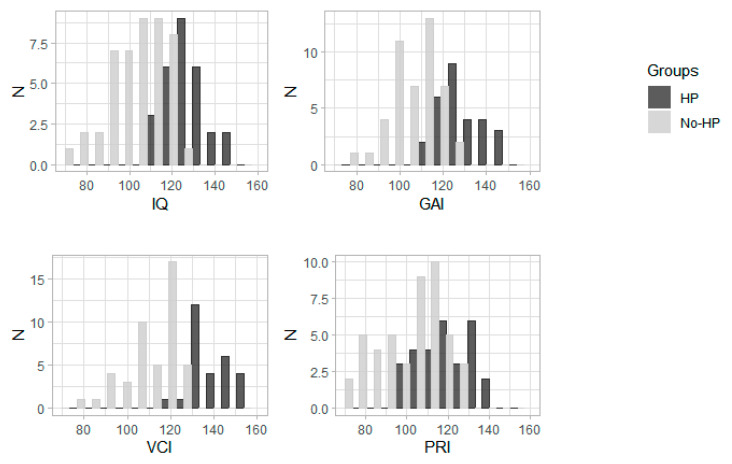
Histograms of intelligence quotient (IQ), GAI, VCI, and PRI in the two groups of children (HP and No-HP).

Children were separated into two groups according to their IQ: 28 children with HP and 46 children without HP (No-HP) (Figure 1). There was no significant difference between groups concerning sex, age, and SEL. In the No-HP group, there were more obese and overweight children than in the HP group but without reaching the significance level. Children with HP reported significantly fewer difficulties at school than No-HP children (Table 1). There was no difference concerning the clinical and electrophysiological characteristics between groups (Table 2 and Table 3). Children with HP had significantly less desaturations ≥3% than children with No-HP. There was no significant difference between HP and No-HP children concerning the Epworth, CDI, or ISI total score. No-HP children tended to have a higher Conners total score, especially, they had more conduct disorders, learning disorders, and impulsivity than those with HP, but no significant difference was found after accounting for multiple comparisons (Table 4). There was no significant difference in the proportion of SVPD between groups (Table 5). The delay between PSG diagnosis and WISC tests was not significantly different between HP and No-HP children (4 vs. 3 months, *p* = 0.47). There were 43 treated patients at the time of the WISC evaluation and no significant difference in the proportion of treated patients nor in the type of therapy (mono vs. bi-therapy) between the HP and No-HP groups.

**Figure 2:**
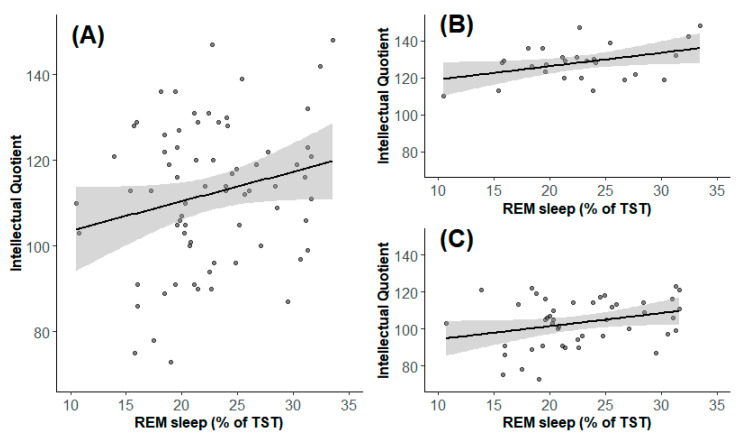
Correlation between IQ and the percentage of REM in the entire group of children with narcolepsy (**A**), in children with HP (**B**) and in children without HP (**C**). Each point represents the percentage of REM sleep for each subject according to the IQ. IQ: Intellectual quotient; REM sleep: Rapid eye movement sleep; HP: High potential.

There was a significant correlation between IQ and the percentage of REM sleep in children with narcolepsy (*r* = 0.25, *p* = 0.04) (Figure 2). The higher the IQ, the higher the REM sleep percentage of TST. There was a trend for this correlation in children with HP (*r* = 0.37, *p* = 0.06) and in those with No-HP (*r* = 0.29, *p* = 0.05).

The multivariate regression model using percentage of REM sleep, BMI-z score, index of desaturations ≥3%, and group (HP vs. No-HP) as independent variables “explained” 52% of the IQ variability. The model explains that an increase of 1% in REM sleep is associated to an increase of the IQ by 0.58 points (*p* = 0.02, 95% confidence interval 0.09–1.06). The same trend between IQ and the percentage of REM sleep also appeared in HP (with an increase of 0.54 points of IQ, *p* = 0.08) and in No-HP (with an increase of 0.62 points of IQ, *p* = 0.11) children but without reaching the significance threshold. At the multivariate level, there was a significant IQ difference between groups (*p* < 0.001), but there was no significant IQ difference depending on the BMI z-score (*p* = 0.68) and on the index of desaturations ≥ 3% (*p* = 0.55).

## 4. Discussion

One of the study’s objectives was to investigate whether intellectual ability constitutes a protective factor for school difficulties, behavioral, and mood impairments in our population. Children with narcolepsy with HP reported fewer difficulties at school than those with No-HP. Even if they also tended to have less impulsivity, conduct disorders, and learning disorders than children without HP, no significant difference was found between these children after accounting for multiple testing. This could be due to the small number of children.

High intellectual abilities seem to be a protective factor against school difficulties in children with narcolepsy. Although many definitions exist, Wechsler defined intelligence as an “aggregate or global capacity encompassing cognitive, emotional, and characteristics aspects, that is, the ability of an individual to act purposefully, to think rationally and to deal effectively with his environment”. This could be understood as a cognitive reserve or, in other terms, as an ability to engage alternative brain networks to compensate for neurological pathologies and behavioral impairments [14,36]. Previous research has suggested that mid-to-high intellectual abilities act as a protective factor on mood, cognitive, and social aspects in patients with neurological or psychiatric disorders [14,21,36,37]. These results are consistent with a previous study suggesting that a mid-to-high IQ is associated with less cognitive impairments and less mood disorders in adults with narcolepsy [14]. In the general population, it is also well known that children with a low IQ are at higher risk for cognitive and behavioral disorders [21,38,39]. However, only few studies investigated whether intelligence plays the same role in children with narcolepsy [11,19,20].

Another study’s objective was to describe the intellectual abilities in a large group of children with narcolepsy and to characterize which factors could influence these abilities. In the present cohort of children with narcolepsy, more than one-third of them were found to be HP, whereas the prevalence of HP is estimated to represent 2.3% in the general pediatric population [32,33]. In the literature, normal IQ performance was found in children with narcolepsy. We do not know how to explain this difference.

The neuropsychological tests used to evaluate intelligence varied in previous studies concerning both adults and children [19,20]. However, compared to other studies (12, 13, and 38 patients) [11,19,20], the present sample of children is larger.

The mid-to-high SEL of the parents herein could represent a recruitment bias. Although the present study took place in one of the two public health national reference centers for children with narcolepsy in France, which recruits half of the children with narcolepsy in this country, families with a mid-to-high SEL could be more likely to consult in such a reference center [40]. It is well known that high potentiality has been previously related to a better SEL [41]. However, no significant difference for SEL was found herein between children with and without HP.

Other factors could also contribute to influence the cognitive profiles of these patients such as the disease’s severity, presence of cataplexies, obesity, diagnosis delay, and presence of treatment. Nevsimalova et al. found that a large proportion of university graduates with narcolepsy with cataplexy had lower Epworth score, fewer sleep attacks, and were usually without treatment compared to those with lower levels of education [7]. In our study, more than half of the children had psychostimulant treatments, which has been shown to improve cognitive performances, especially alertness and executive functions [42,43,44]. However, the same proportion of patients treated with mono and bi-therapy was found in children with HP and No-HP. Ingravello et al. reported that the level of education attained by patients with narcolepsy was above that of the Italian population [45]. These findings are consistent with those of the few studies that have investigated this topic, highlighting that people with narcolepsy achieve an equivalent educational level to those without and had an overall high educational level [7,44,46]. These studies assumed that narcolepsy per se does not prevent the achievement of a good educational level, reflecting the good cognitive profile of these patients.

Hypocretin deficit could also be implicated in the cognitive performance found in patients with narcolepsy type 1. Hypocretin-1 system has widespread interactions with the brain’s neurotransmitters network, including the prefrontal cortex involved in executive and attentional functioning [11]. Knowing that narcolepsy with cataplexy is caused by a deficit in hypocretin-1 cells, these circuits might be destabilized, causing sleepiness, attention, and executive disorders. However, no difference was found between narcoleptic children with and without HP for the disease characteristics.

Compared to children with HP, children with No-HP were more frequently obese and were thus more at risk of obstructive sleep apnea (OSA), which could be reflected by their higher desaturation indexes. We can hypothesize that children without HP have more repercussions of their obesity on their gas exchanges (reflected by desaturations) than those with HP, even if it is not sufficiently severe to impact their minimal saturation. It was previously related that OSA in children might produce cognitive and behavioral sequelae [19,47]. This could indicate that the No-HP group represents a more severe expression of the disease. Obesity is found in more than 50% of children with narcolepsy [8,48]. In our study, we did not find a statistically significant difference for obesity/overweight between groups after corrections for multiple comparisons, which was probably due to our limited number of patients. The mechanisms responsible for obesity in children with narcolepsy are not well known and could involve a severe hypothalamic involvement, decreased metabolism, and subtle changes in eating behavior [8,48].

Interestingly, we found a positive correlation between the percentage of REM sleep and IQ in children with narcolepsy. Indeed, the higher the percentage of REM sleep, the higher the IQ. These results are concordant with a previous study from our team suggesting that children with HP have more REM sleep than children with a normal IQ [35]. The loss of excitatory hypocretin neurons reduces the activity of the GABA neurons of the ventrolateral periaqueductal gray matter in the midbrain, which trigger the presence of REM sleep in patients with narcolepsy [49]. REM sleep is implicated in memory consolidation, learning, and creativity [50,51,52]. REM sleep could herein be involved in the dynamic changes contributing to the equilibrium of intellectual functioning between genes, brain, behavior, cognition, and environment [53].

In the other hand, we might ask if individuals with superior IQ could be at risk to develop narcolepsy. Individuals of superior IQ (compared to average and high) show more intense and prolonged frontal and parietal cortical thickening, followed by more rapid thinning. This distinct trajectory may reflect prolonged synaptogenesis and an extended sensitive period, during which the brain is particularly responsive to environmental input [54,55]. Despite extensive research, the etiology of narcolepsy is still unclear. Narcolepsy with cataplexy is a multifactorial disease caused by both genetic and environmental factors. Several genetic factors including HLA-DR-DQB1*06:02 or other new narcolepsy-associated genes have been identified [56]. Genetic variation in the HLA region has been associated with cognitive ability in pathological contexts [57,58]. However, since all the patients in the present study were positive for the HLA-DR-BQB1*06:02, this factor alone is unlikely to explain the high incidence of HP. The HLA-DR-DQB1*06:02 variant could provide a sensitized genetic background for other genetic and epigenetic factors such as DNA methylation, which have been suggested to play an important role in the pathogenesis of complex diseases such as narcolepsy [59]. In addition, environmental factors such as exposure to H1N1 ASO3 adjuvanted pandemic vaccine or streptococcus pyogenes infections could interfere in the pathophysiology of narcolepsy [56].

The present study has some limitations.

The first limitation is due to the design: a cross-sectional study cannot infer the direction of the relationship between IQ and sleep characteristics, and no causality can be drawn. Moreover, the addition of a control group composed by healthy children with and without HP could have allowed us to distinguish the effects related to HP from those related to narcolepsy itself. Second, we could assume that these results concerned children with narcolepsy type 1, but not all children with narcolepsy had cataplexy and/or CSF hypocretin measurements in our study. However, among the seven children without cataplexy, four had CSF hypocretin measurements, and all of them were hypocretin deficient (<50 pg/mL). Third, a majority of children were under treatment, which may improve their cognitive abilities. Indeed, animal models of hypocretin deficiency suggest that the pathology could be associated with a deficit of dopamine, which may be at least partially corrected by a treatment such as methylphenidate [60]. However, we did not find an IQ difference between patients with and without treatment. Fourth, if the relationship between the REM sleep percentage and the IQ was significant, the value of the correlation was low. Moreover, only one-night PSG was recorded, and it may not reflect the usual sleep characteristics of children. If these results must be considered knowing this aspect, it is interesting to highlight that we found the same correlation in healthy children with and without HP [35]. Another limitation is that we do not report information about parents IQ or educational levels, which are two factors that play a major role in both the genetic and hereditary characteristics of children’s IQ. Moreover, our analysis concerns only the sleep macrostructure; it would be interesting to study the microstructure of sleep by spectral analysis [61] to deepen our understanding of the relation between sleep and cognitive performances. In the present study, the evaluation of depressive feelings, attention, and school difficulties were done by questionnaires, it would be interesting to evaluate them objectively and further to assess the relation between the cognitive performances and the social and professional integration of these children in adult life [7]. Finally, even with a normal-to-high IQ, children with narcolepsy have school difficulties. Herein, attentional and executive performances were not especially assessed at the time of the test. It would be appropriate to supplement the assessment of IQ with other neuropsychological tests of specific cognitive functions.

## 5. Conclusions

The present results suggest that narcolepsy in children is unlikely to be a cause of intellectual disability despite their frequent school difficulties. Neuropsychological evaluation could help these children to find adapted support. The prompt diagnosis and management of comorbidity such as obesity and OSA could improve cognitive performances and decrease school and behavioral difficulties in these children.

## Figures and Tables

**Table 1:** Demographic characteristics of children with and without high potential (HP).

	Global	*n*	HP	*n*	No-HP	*n*	*p*
		74		28		46	
Age, years	11.5 (5.5–17.4)	74	11.3 (6.6–17.1)	28	11.7 (5.5–17.4)	46	0.87
Sex, male % (*n*)	58 (43)	74	46 (13)	28	65 (30)	46	0.61
BMI z-score	2.8 (−1.8–9)	73	2.2 (−0.5–7.6)	28	3.3 (−1.8–9)	45	0.18
BMI z- score classification		73		28		45	0.28
*Normal, % (n)*	26 (19)		43 (12)		16 (7)		
*Obesity, % (n)*	64 (47)		50 (14)		73 (33)		
*Overweight, % (n)*	10 (7)		7 (2)		11 (5)		
SEL, % (*n*)		66		26		40	0.64
*Farmers*	3 (2)		4 (1)		3 (1)		
*Artisans, shopkeepers, CEOs*	5 (3)		4 (1)		5 (2)		
*Executive and intellectual professions*	20 (13)		31 (8)		12 (5)		
*Intermediate professions*	24 (16)		27 (7)		22 (9)		
*Employees*	27 (18)		27 (7)		28 (11)		
*Workers*	6 (4)		0 (0)		10 (4)		
*Students and unemployed*	15 (10)		7 (2)		20 (8)		
School difficulties, % (*n*)	48 (34)	71	21 (6)	28	65 (28)	43	0.01

Values are reported as median (range) except otherwise indicated. BMI: Body mass index; SEL: Socioeconomic level.

**Table 2:** Narcolepsy characteristics in children with and without HP.

	Global	*n*	HP	*n*	No-HP	*n*	*p*
		74		28		46	
Age at diagnosis, years	11.7 (5.5–17.6)	74	11 (6.7–7.5)	28	12 (5.5–17.4)	46	0.86
Age at onset, years	9.6 (4.2–15.8)	71	10 (5.9–15.6)	28	8.5 (4.2–15.8)	43	0.54
H1N1 vaccine, % (*n*)	23 (13)	56	33 (7)	21	17 (6)	35	0.61
Cataplexies, % (*n*)	91 (67)	74	96 (27)	28	87 (40)	46	0.61
Sleep paralysis, % (*n*)	18 (13)	74	18 (5)	28	17 (8)	46	1.00
Hypnagogic hallucinations, % (*n*)	39 (29)	74	39 (11)	28	39 (18)	46	1.00
HLA +, % (*n*)	100 (71)	71	100 (28)	28	100 (43)	43	1.00
Hypocretin, pg/mL	21 (0–178)	40	20 (0–178)	11	22 (1–90)	29	0.71

Values are reported as median (range) except otherwise indicated.

**Table 3:** Sleep and respiratory characteristics of children with and without HP.

	Global	*n*	HP	*n*	No-HP	*n*	*p*
PSG				28		46	
Total sleep time, min	478 (270–615)	72	463 (324–561)	27	485 (270–615)	45	0.45
Sleep efficiency, %	84.1 (52.5–95.2)	71	83.7 (56.6–91.4)	27	84.1 (52.5–95.2)	44	0.54
Sleep latency, min	4.8 (0–78)	72	6 (0–78)	27	4 (0–77)	45	0.64
REM latency, min	5.8 (0–225)	72	10.3 (0–225)	27	4.5 (0–215.5)	45	0.86
Stage 1, %	15.3 (0.2–32)	72	16 (0.2–28.3)	27	15.3 (2.8–32)	45	0.90
Stage 1, min	67 (1–173)	72	64 (1–137)	27	68 (11–173)	45	0.64
Stage 2, %	41.4 (17.4–64.7)	72	39 (26.3–64.7)	27	42.4 (17.4–56.7)	45	0.64
Stage 2, min	191 (23.5–293)	72	178 (23.5–293)	27	201 (72–291)	45	0.35
Stage 3, %	19.6 (0.2–59.5)	72	19.8 (9.1–34.6)	27	19.4 (0.2–59.5)	45	0.55
Stage 3, min	94 (1–246)	72	93 (28.2–145.5)	27	95 (1–246)	45	0.94
REM sleep, %	22.3 (10.5–39.3)	72	22.7 (10.5-39.3)	27	21.4 (10.7–31.6)	45	0.90
REM sleep, min	110 (17.9–190)	72	102 (17.9-190)	27	112.5 (29–178)	45	0.54
Total arousal index, %	11.8 (0–66.6)	69	12.3 (6.2-66.6)	26	11.5 (0–37.4)	43	0.90
Desaturation ≥3% index, %	0.05 (0–19.3)	68	0 (0–1.5)	25	0.4 (0–19.3)	43	0.01
Minimal saturation, %	93 (30–98)	70	92.8 (69.3–97)	25	93 (30–98)	45	0.88
OAHI	0.5 (0–7.5)	68	0.40 (0-3.6)	26	0.55 (0–7.5)	42	0.54
MSLT							
MSLT, *n*	4 (4,5)	74	4 (4,5)	28	4 (4,5)	46	0.59
SOREM, %	100 (25–100)	74	100 (25–100)	28	100 (25–100)	46	0.97
Sleep latency, min	2.5 (0–10)	74	2 (0–10)	28	3.3 (0.4–8)	46	0.54

Values are reported as median (range) except otherwise indicated. PSG: Polysomnography; REM: Rapid eye movement; OAHI: Obstructive apnea hypopnea index; MSLT: Multiple sleep latency test; SOREM: Sleep onset in REM.

**Table 4:** Questionnaire scores in children with and without HP.

	Global	*n*	HP	*n*	No-HP	*n*	*p*
Epworth total score	17 (9–23)	71	16 (9–23)	27	18 (9–23)	44	0.63
Epworth pathologic score, % (*n*)	96 (68)	71	96 (26)	27	96 (42)	44	1.00
ISI total score	12 (2–22)	62	12 (4–22)	23	12 (2–21)	39	0.86
ISI pathologic score, % (*n*)	71 (44)	62	70 (16)	23	72 (28)	39	1.00
CDI total score	10 (0–38)	62	9 (0–38)	25	10 (2–30)	37	0.63
CDI pathologic score, % (*n*)	21 (13)	62	20 (5)	25	22 (8)	37	1.00
Conners Total Score	18 (1–58)	54	14.5 (1–58)	20	21 (1–44)	34	0.24
Conners pathologic score, % (*n*)	0 (0)	54	0 (0)	20	0 (0)	34	1.00
*Conduct disorders*	49 (9–92)	54	43.5 (39–92)	20	50 (9–78)	34	0.20
*Learning disorders*	57 (36–107)	54	45 (36–98)	20	57 (38–107)	34	0.18
*Psychosomatization*	53 (42–106)	54	45 (42–106)	20	53 (42–83)	34	0.62
*Impulsivity*	46 (35–75)	54	43 (35–68)	20	47 (35–75)	34	0.24
*Anxiety*	50 (39–78)	54	51 (40–78)	20	49 (39–68)	34	0.63
*Hyperactivity*	52 (33–88)	54	48.5 (35–88)	20	54 (33–76)	34	0.41

Values are reported as median (range) except otherwise indicated. ISI: Insomnia severity scale; CDI: Children depression inventory.

**Table 5:** WISC characteristics in children with and without HP.

	Global	*n*	HP	*n*	No-HP	*n*	*p*
VCI	118 (76–155)	74	136.5 (118–155)	28	113 (76–128)	46	<0.0001
PRI	109 (72–142)	74	116 (99–142)	28	103 (72–128)	46	<0.0001
WMI	103 (58–130)	65	109 (76–130)	25	95.5 (58–118)	40	<0.0001
PSI	100 (64–151)	72	112 (76–151)	27	96 (64–124)	45	0.006
GAI	115 (80–148)	74	128 (115–148)	28	107 (80–123)	46	<0.0001
IQ	114 (73–148)	74	128.5 (110–148)	28	105 (73–123)	46	<0.0001
SVPD, % (*n*)	18 (13)	74	25 (7)	28	13 (6)	46	0.61
Treatment, % (*n*)	58 (43)	74	57 (16)	28	59 (27)	46	1.00
Therapy type		74		28		46	1.00
*None*	42 (31)		43 (12)		41 (19)		
*Monotherapy*	41 (30)		39 (11)		41 (19)		
*Bi-therapy*	17 (13)		18 (5)		17 (8)		

Values are reported as median (range) except otherwise indicated. VCI: Verbal comprehension index; PRI: Perceptual reasoning index; WMI: Working memory index; PSI: Processing speed index; GAI: General abilities index; IQ: Intelligence quotient; SVPD: Significant verbal performance discrepancy.

## Abbreviations

(BMI)	Body mass index
(CDI)	Child depression inventory
(GAI)	General aptitude index
(HP)	High potential
(ISI)	Insomnia severity index
(IQ)	Intelligence quotient
(MSLT)	Multiple sleep latency test
(OAHI)	Obstructive apnea hypopnea index
(OSA)	Obstructive sleep apnea
(PRI)	Perceptual reasoning index
(PSG)	Polysomnography
(PSI)	Processing speed index
(REM)	Rapid eye movement
(SVPD)	Significant verbal performance discrepancy
(VCI)	Verbal comprehension index
(WISC)	Wechsler Intelligence Scale for Children
(WMI)	Working memory index
